# Exacerbation of Repetitive Falls Due to Atonic Seizures Following Perampanel Administration

**DOI:** 10.7759/cureus.40818

**Published:** 2023-06-22

**Authors:** Akiko Maeda, Shuichiro Neshige, Riho Katsumata, Megumi Nonaka, Haruka Ishibashi, Hirofumi Maruyama

**Affiliations:** 1 Department of Clinical Neuroscience and Therapeutics, Hiroshima University Graduate School of Biomedical and Health Sciences, Hiroshima, JPN; 2 Department of Clinical Neuroscience and Therapeutics, Hiroshima University, Hiroshima, JPN

**Keywords:** perampanel, atonic seizures, drug-resistant epilepsy, lennox-gastaut syndrome, cognition, neurology, neurophysiology, seizure medications, electroencephalography (eeg)

## Abstract

A 47-year-old man presented with tonic-clonic seizures characterized by convulsions. He repeatedly exhibited seizures despite treatment with four anti-seizure medications. During the titration process of perampanel (PER), the seizures paradoxically increased in intensity and frequency, resulting in trauma. Video electroencephalogram monitoring revealed interictal rapid rhythms and generalized spikes and documented atonic seizures. Thus, the patient was diagnosed with Lennox-Gastaut syndrome. Upon discontinuation of PER, the patient’s atonic seizures with falls improved, probably suggesting a paradoxical effect of PER. A non-competitive antagonist selective for AMPA (α-amino-3-hydroxy-5-methyl-4-isoxazolepropionic acid) receptors may have caused the weakness and delayed recovery from prolonged atonia that caused injuries.

## Introduction

Paradoxical effect (PE) is a phenomenon in which a paradoxical exacerbation of seizures occurs after the introduction of an anti-seizure medication (ASM) that was effective at the time of its introduction [1]. Seizure exacerbation may include increased seizure frequency or the appearance of a new type of seizure. It should be noted that PE can be visible with any kind of ASM, including conventional and novel drugs, such as carbamazepine (CBZ) [2], levetiracetam (LEV) [3-7], and lamotrigine (LTG) [8-11].

Recently, PE was also reported in a case of perampanel (PER) [12,13]. However, the underlying mechanisms and clinical features of PE in PER are still limited. Here, we report an adult case who was diagnosed with Lennox-Gastaut syndrome (LGS) with myoclonic and atonic seizures. The patient exhibited increases in the frequency and intensity of seizures during the titration process of PE, probably suggesting a PE of PER.

## Case presentation

**Figure 1:**
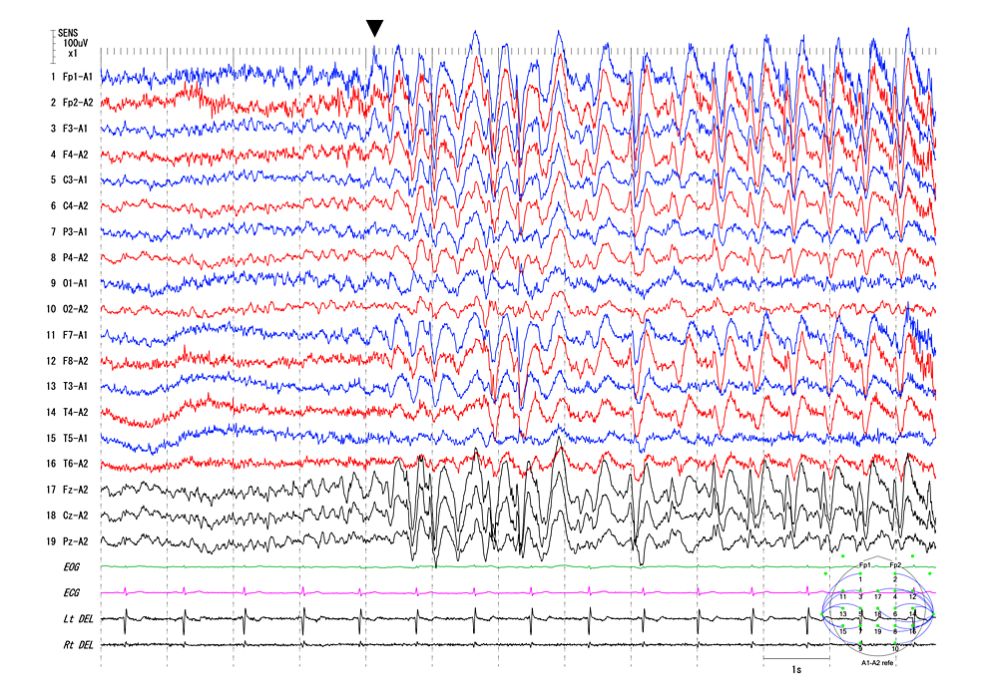
Interictal EEG findings The EEG was recorded at a sampling rate of 500 Hz. Generalized spike slow wave complex at 1.5 to 2.5 Hz. Arrowhead indicates the onset of spike. EOG: electrooculogram; ECG: electrocardiogram; Lt and Rt DEL: left and right deltoid electromyogram.

**Figure 2:**
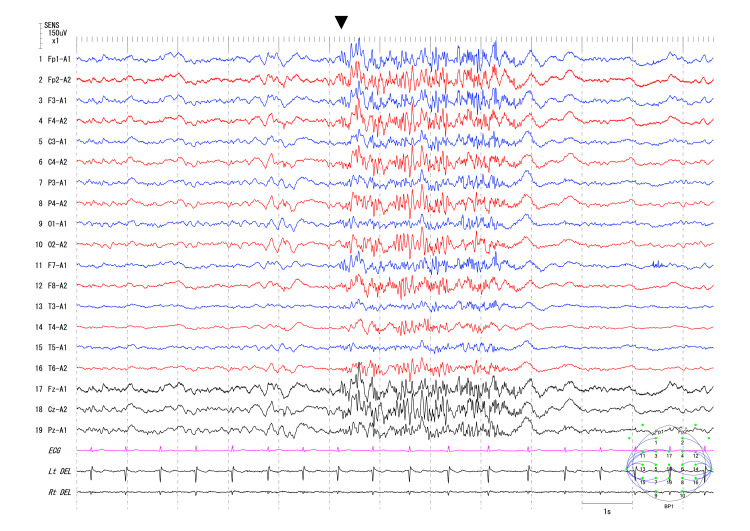
Rapid rhythm Arrowhead indicates the onset of rapid rhythm. ECG: electrocardiogram; Lt and Rt DEL: left and right deltoid electromyogram.

The patient was a 47-year-old male with no history of precipitating injuries, such as central nervous system infection, febrile convulsions, head injury, perinatal abnormalities, or a family history of epilepsy. There were no developmental abnormalities. He exhibited seizures with falls for a few weeks and infrequent tonic-clonic seizures at four years of age. In his 20s, he was diagnosed with frontal lobe epilepsy and treated with four ASMs (valproic acid (VPA), phenobarbital (PB), LTG, and CBZ). Despite the treatment, the patient repeatedly experienced falls with trauma, including jaw fractures, approximately, four to five times per year. Consequently, he visited our department at the age of 47 years. Granulation bumps and crusting were observed on both knee joints due to frequent traumatic injuries. Neurological examination revealed mild mental retardation, with seizures presenting as impairments of awareness, weakness in limbs with falling (five to six times/month, lasting two to three minutes), and myoclonic or tonic seizures (one to two times/month, lasting for a few seconds). The blood levels of VPA, PB, LTG, and PER were 58.8 μg/mL, 30.7 μg/mL, 4.4 μg/mL, and 197 ng/mL, respectively, which were all within the optimal range (under treatment with VPA 2400 mg/day, PB 120 mg/day, LTG 150 mg/day, and PER 6 mg/day). Brain magnetic resonance imaging showed diffuse brain atrophy without evidence of hippocampal sclerosis or cortical dysplasia. Interictal electroencephalogram (EEG) showed generalized spikes and a slow wave complex at 1.5 to 2.5 Hz (Figure 1), and rapid rhythm (Figure 2).

**Figure 3:**
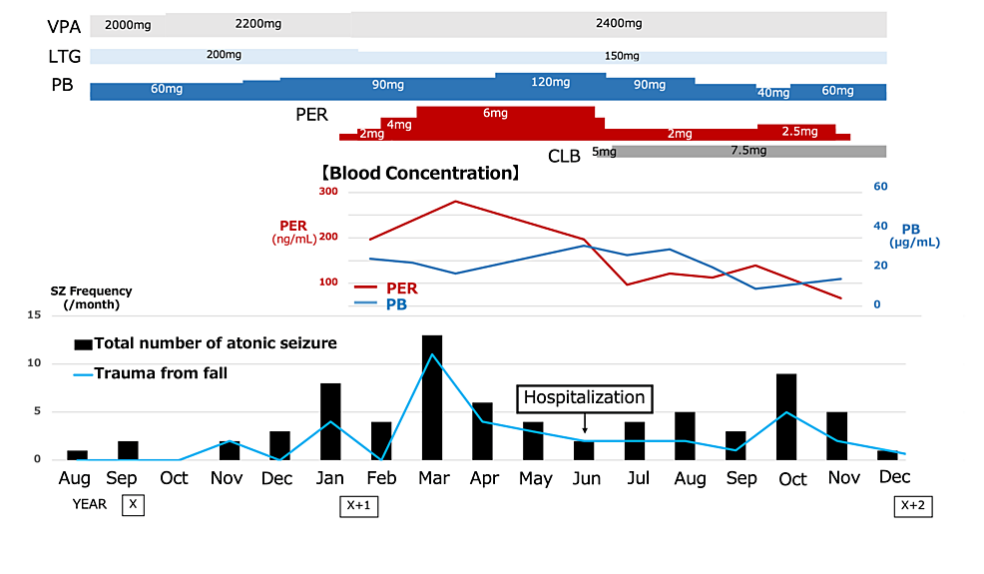
Clinical course The changes in the dose and blood concentration of the anti-seizure medications are shown according to the change in seizure frequency and injury severity. A positive correlation between seizure frequency (and injury severity) and PER concentration was observed two times. In contrast, an inverse correlation was observed between PER and PB blood concentrations. Carbamazepine had been discontinued prior to the duration of the clinical course depicted in this figure. VPA: valproic acid; LTG: lamotrigine; PB: phenobarbital; PER: perampanel; CLB: clobazam; Sz: seizure.

Due to the presence of myoclonic seizures in generalized epilepsy, CBZ was discontinued. Nine months after the discontinuation, PER was initiated at a dose of 2 mg, which was subsequently titrated to 6 mg. Despite the initial mild effect of PER, the frequency of seizures and incidents of falls resulting in injury paradoxically increased during the titration period, peaking at 6 mg/day, along with an increase in the blood concentration of PER (Figure 3). Consequently, the dose of PB was augmented. Thereafter, a marginal decrease in the frequency of seizures was noted. A corresponding alteration in the blood levels of PER (decline) and PB (increase) was also visible. Furthermore, the dosage of PER was reduced to 2 mg, and clobazam was introduced. Subsequently, a gradual decline was observed in both the overall number of atonic seizures and the frequency of falls-related traumatic injuries, in conjunction with a reduction in PER blood concentration (Figure 3). However, with the re-escalation of the PER dosage, the frequency of seizures and the incidence of trauma substantially increased. Consequently, following PER discontinuation, the frequency of seizures reverted to baseline levels, and no severe traumatic injuries were observed thereafter.

**Figure 4:**
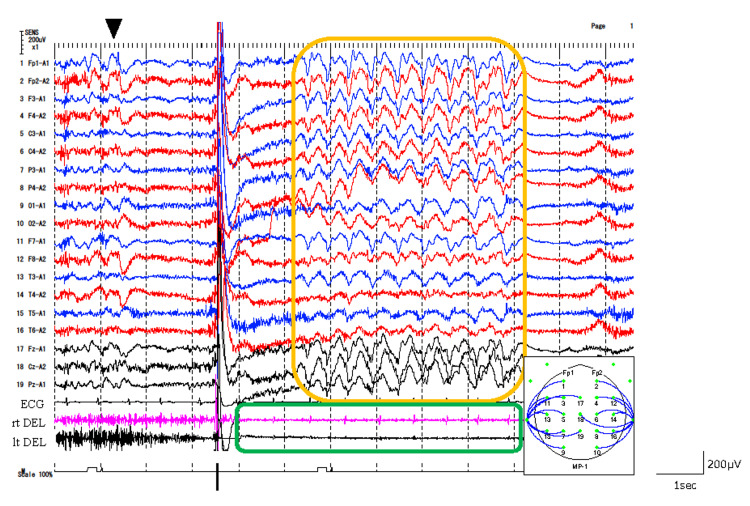
Ictal EEG The video EEG monitoring test supplemented the seizures of sudden weakness and backward falling. The ictal EEG shows a bifrontal predominant generalized spike (black arrowhead) followed by background attenuation, a 1.5 to 2 Hz spike, and a slow wave complex (yellow area). After the ictal onset, atonia was observed in the bilateral limbs with electromyography (green area). DEL: deltoid muscle; EEG: electroencephalogram; ECG: electrocardiogram.

The patient underwent video EEG monitoring to ascertain why seizures occurred causing weakness and falling backward. Ictal EEG revealed bilateral frontally predominant generalized spikes, followed by background attenuation, low amplitude electromyography, and 1.5 to 2 Hz spike and slow wave complexes (Figure 4). Therefore, he was diagnosed with LGS.

## Discussion

LGS is a neurologically severe condition characterized by drug-resistant epilepsy with tonic, atonic, atypical absence, myoclonic, and generalized tonic-clonic seizures [14]. The patient in the present case lacked atypical absence seizures. Nonetheless, the diagnosis of LGS was established based on the presence of atonic and myoclonic seizures and a generalized rapid rhythm in the EEG examination, which is a hallmark of the disease.

PER is utilized in patients with focal seizures, including secondarily generalized seizures and tonic-clonic seizures, and is frequently employed in the treatment of drug-resistant epilepsy [15]. In the present case, following the discontinuation of CBZ, the frequency of seizures decreased while the patient was receiving VPA, LTG, and PB. However, PER initiation was necessitated by the presence of residual seizures. After the introduction of PER, the frequency and intensity of seizures worsened as the blood concentration of PER increased, but both decreased as the blood concentration declined. Because the paradoxical worsening of seizures was reproducibly confirmed when the PER was titrated two times, we considered that the time course of medication and seizure frequency was consistent with the presentation of PE. However, the effects of changing other drugs, such as clobazam (CLB), also need to be considered due to the change in seizure frequency. Although CLB might have been effective, the correlation between the blood PER and PB levels and seizure frequency supported that the patient had PE with PER.

Though the precise mechanism underlying PE remains unknown, risk factors for PE have been suggested, including intractable focal epilepsy, a relatively young age, and multiple drug use [3-7]. Nevertheless, the incidence of PE in PER remains limited. In a study of idiopathic generalized epilepsy with refractory tonic-clonic seizures, it was reported that the administration of 8 mg of PER resulted in an exacerbation of myoclonic seizures in 22.2% of subjects treated with PER, compared with 12.1% of subjects receiving a placebo [12,13]. In another study of patients with drug-resistant focal epilepsy, 28.6% discontinued PER due to PE [12,13], and in a study of patients with drug-resistant epilepsy, including both generalized and focal seizures, worsening of seizures and PE were reported in 9% of patients after PER administration [16]. However, it is worth noting that these reports did not investigate cases of atonic seizures or LGS. Thus, this is the first report of PE caused by PER associated with these phenotypes. Besides, it should be noted that there have been several reports that perampanel is effective and well-tolerated in LGS [17,18]. The clinical presentation of our case with LGS was primarily characterized by atonic seizures rather than tonic seizures. Thus, we have postulated that the paradoxical effect of the administered PER medication might have been more prominent than its therapeutic effectiveness in managing this specific syndrome.

In the present case, we considered that seizure worsening had two facets. The first is the exacerbation of seizures as a presentation of typical PE [16]. The second is an increase in the intensity of the injury (worsening of the degree of trauma caused by the seizures). PER is a non-competitive antagonist selective for α-amino-3-hydroxy-5-methyl-4-isoxazolepropionic acid (AMPA)-type glutamate receptors that decrease brain activity due to the inhibition of glutamate receptors [19]. We speculate that such inhibition may have caused the weakness and delayed recovery from prolonged atonia observed after seizures, particularly atonic seizures. Thus, a weakened state during atonic seizures and delayed recovery from this state may have intensified the degree of injury sustained during seizures, resulting in severe injuries. Therefore, dose escalation of PER may need to be performed with caution in patients with atonic seizures. Clinicians usually discontinue CBZ when patients experienced myoclonic and generalized seizures. Thus, confirming whether a patient has atonic seizures would be beneficial before introducing PER in such a situation. Besides, given that the frequency of seizures was also increased following the PER administration, there might be a chance that subclinical atonic seizures were enhanced and developed as "clinical seizures."

## Conclusions

Clinicians ought to bear in mind that there exist individuals with LGS with atonic seizures who go undiagnosed until reaching adulthood. We confirmed the exacerbation of atonic seizures with repetitive falls during PER dose titration in an adult patient with LGS. This may hold true in cases of LGS that predominantly present with atonic seizures instead of tonic seizures. An atonic seizure may be a red flag sign of PE caused by PER that substantially inhibits glutamate receptors.
